# Microstructure, Mechanical, and Tribological Properties of Nb-Doped TiAl Alloys Fabricated via Laser Metal Deposition

**DOI:** 10.3390/ma17174260

**Published:** 2024-08-28

**Authors:** Kai Huang, Feng Xu, Xinyan Liu, Shiqiu Liu, Qingge Wang, Ian Baker, Min Song, Hong Wu

**Affiliations:** 1 State Key Laboratory of Powder Metallurgy, Central South University, Changsha 410083, China; 2 Farsoon Technologies Co., Ltd., Changsha 410221, China; 3 Advanced Materials Additive Manufacturing Innovation Research Center, School of Engineering, Hangzhou City University, Hangzhou 310015, China; 4 Thayer School of Engineering, Dartmouth College, Hanover, NH 03755, USA

**Keywords:** TiAl alloys, Nb alloying, laser metal deposition, microstructure, mechanical properties, tribological properties

## Abstract

TiAl alloys possess excellent properties, such as low density, high specific strength, high elastic modulus, and high-temperature creep resistance, which allows their use to replace Ni-based superalloys in some high-temperature applications. In this work, the traditional TiAl alloy Ti-48Al-2Nb-2Cr (Ti4822) was alloyed with additional Nb and fabricated using laser metal deposition (LMD), and the impacts of this additional Nb on the microstructure and mechanical and tribological properties of the as-fabricated alloys were investigated. The resulting alloys mainly consisted of the γ phase, trace β_0_ and α_2_ phases. Nb was well distributed throughout the alloys, while Cr segregation resulted in the residual β_0_ phase. Increasing the amount of Nb content increased the amount of the γ phase and reduced the amount of the β_0_ phase. The alloy Ti4822-2Nb exhibited a room-temperature (RT) fracture strength under a tensile of 568 ± 7.8 MPa, which was nearly 100 MPa higher than that of the Ti4822-1Nb alloy. A further increase in Nb to an additional 4 at.% Nb had little effect on the fracture strength. Both the friction coefficient and the wear rate increased with the increasing Nb content. The wear mechanisms for all samples were abrasive wear with local plastic deformation and oxidative wear, resulting in the formation of metal oxide particles.

## 1. Introduction

The intermetallic compound TiAl, which has a density of ~4000 kg/m^3^, exhibits exceptional mechanical and chemical properties at high temperatures, including high specific strength, high specific modulus, substantial creep resistance, considerable fatigue resistance, and excellent oxidation resistance [1,2,3]. TiAl alloys are considered the most promising candidates to replace Ni-based superalloys within the temperature range of 650~850 °C [4,5]. They have been used effectively in racing engine parts, aero-engine low-pressure turbine (LPT) blades, and in several other applications [6,7], and enable a reduction in structural weight, thereby decreasing pollutant emissions while enhancing energy efficiency [8,9]. Between 2008 and 2018, the overall manufacturing expenses for γ-TiAl alloys at the German Electrometallurgy Company dropped by almost 70%, while production surged almost thirtyfold, demonstrating their increased commercial use [10,11,12]. Presently, the main processing technologies for TiAl alloys include ingot metallurgy, precision casting, and powder metallurgy [13,14,15]. However, both their limited hot processing range and low room-temperature plasticity significantly hinder their widespread industrial use. The ingot metallurgy process is complex and encompasses melting, casting, hot isostatic pressing, and hot processing, resulting in a high rate of defective products and high costs due to significant chemical segregation [16,17]. Although LPT blades produced by precision casting have been used on a small scale, it remains challenging to manufacture components with intricate internal cavity structures [18]. On the other hand, powder metallurgy is mostly confined to laboratory research, and faces limitations regarding the size and shape of components [19].

In the past two decades, laser additive manufacturing (LAM) has emerged as the most rapidly evolving and promising technology in advanced manufacturing [20,21,22,23,24]. As one of the exemplary representatives of LAM technology, laser metal deposition (LMD) technology possesses distinctive features such as high energy density, exceptional processing precision, shortened processing cycles, and customized production capabilities [25,26]. Moreover, LMD technology transcends the limitations imposed by molds and size restrictions to directly manufacture parts with intricate shapes [27,28]. This enhances the design flexibility for new products while simultaneously reducing manufacturing costs [29,30].

The components created by LMD show complex microstructures and mechanical properties, which are notably different from those of conventionally cast or forged parts [31,32]. This is a result of the distinctive thermal conditions encountered throughout the LMD process, including both rapid heating (10^6^–10^7^ °C∙s^−1^) and cooling (>10^3^ °C∙s^−1^) cycles [33,34]. TiAl alloys are brittle, and thus, susceptibility to cracking is a major challenge during their LMD processing. Recently, researchers have successfully prepared crack-free TiAl alloy blocks by adjusting the processing parameters, e.g., the laser power and auxiliary heating, to reduce cooling rates [35,36].

Most research on TiAl alloys made by LMD has concentrated on the traditional alloy Ti-48Al-2Nb-2Cr (Ti4822). For instance, Wang et al. [37] prepared Ti4822 blocks using high-power LMD that had an ultimate tensile strength (UTS) of 545 ± 9 MPa at ambient temperatures and 471 ± 37 MPa at 760 °C. The microstructure consisted of alternating columnar and equiaxed grains. Wu et al. [28] also observed this phenomenon and explained the evolution of the microstructure and the resulting change in hardness. Zhang et al. [38] studied the anisotropy of the tensile behavior of the closely related alloy Ti-47Al-2Nb-2Cr produced by LMD and obtained a UTS at a room temperature of ~650 MPa. Ti4822 is a composition system suitable for casting, but it is not obvious that it is suitable for LMD processing, which has high cooling rates and cyclic heating characteristics [39,40]. Thus, alloying elements may exhibit different effects in LMD processing compared to processing using conventional casting. Therefore, it is essential to investigate the impacts of various alloying elements on LMD-processed alloys. This will provide a basis for designing specific alloy compositions for LMD.

Nb is commonly used as a β stabilizer in TiAl alloys and extends the β-phase region to a high Al content [41,42]. This leads to an elevation in both the melt temperature (T_m_) and eutectoid temperature (T_eu_), as well as a shift of the solute temperature for the γ phase towards the Al-rich side [43]. These modifications have significant implications for refining microstructures. In addition, the addition of Nb can greatly improve the high-temperature mechanical properties via solid solution strengthening, while also enhancing oxidation resistance [44,45]. The importance of incorporating Nb has increased significantly since the development of the second generation of TiAl alloys, particularly high-Nb TiAl alloys [11]. Hence, it is crucial to explore the impacts of Nb on the fabrication of TiAl alloys via LMD.

In some practical engineering applications, including exhaust valves, turbine blades, and dispersion flaps, sliding contact is necessary for the components during their service life. This requires TiAl alloys to exhibit not only superior strength, but also good wear resistance, which necessitates an understanding of their tribological properties. Previous studies have predominantly concentrated on the friction and wear properties of TiAl alloys with low Nb content prepared through conventional processing. Wang et al. [46] investigated the effect of lamellar orientation on the friction and wear properties of directionally solidified Ti-47Al-2Nb-2Cr-2Mn and found that the highest friction coefficient was observed for a direction of 70–90°, followed by 35–50° and 0–15°, with the wear rate showing the opposite trend. Cheng et al. [47] investigated the tribological behavior of Ti-46Al-2Cr-2Nb alloys prepared by hot-press sintering at different temperatures and found that the coefficient of friction slowly decreased with increasing temperatures. Nevertheless, the friction properties of LMD-ed TiAl alloys are rarely reported.

This study employed LMD to produce TiAl alloys with different Nb contents. The microstructural evolution, tensile properties, hardness, and tribological properties of the alloys were investigated and analyzed. The aim is to offer valuable insights into the compositional design of high-performance TiAl alloys, especially those with excellent tribological properties, processed by LMD additive manufacturing technology.

## 2. Materials and Methods

**Figure 1:**
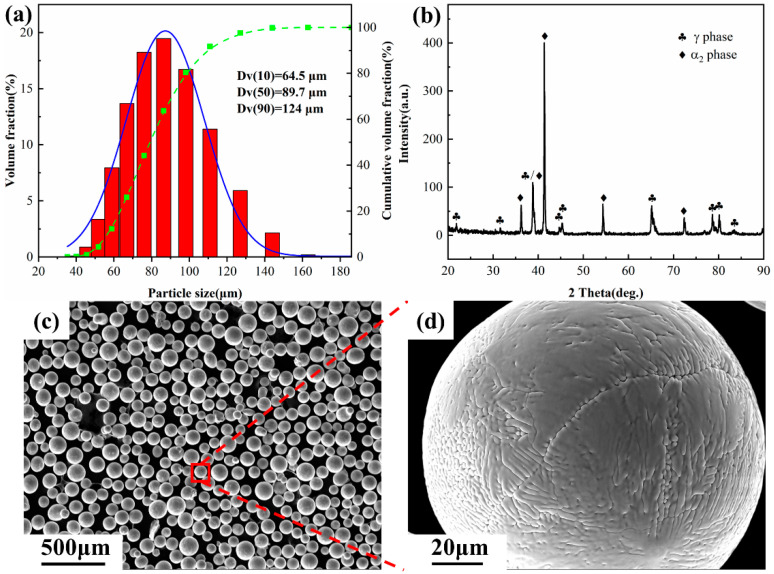
Ti4822 pre-alloyed powder: (**a**) particle size distribution (blue line represents the frequency fraction and green line represents cumulative fraction), (**b**) XRD pattern, (**c**) secondary electron images of the powder, and (**d**) higher magnification image from the red box in (**c**).

Powders of Ti4822 and high-purity Nb (>99.6%, particle size range from 15 to 53 μm, purchased from Bright Laser Inc., Xian, China) were used in this study. The plasma rotation electrode procedure was used to create the Ti4822 powders. As shown in Figure 1a, the resulting particle size distribution, which was characterized by a Mastersizer3000 (Malvern Panalytical, Malver, UK) laser particle size analyzer, spanned 40–150 μm, with Dv(50) = 89.7 μm. X-ray diffraction (XRD) patterns, produced using Cu-K_α_ radiation for a diffraction angle (2θ) ranging from 20 to 90° in increments of 0.02°, were used to determine the phases present in the powder and the processed alloys. The morphology of the Ti4822 powder was characterized using an Quanta FEG250 scanning electron microscope (SEM, FEI, Hillsboro, OR, USA). Three mixed powders with different Nb contents were obtained by mixing the Ti4822 powder with the Nb powder to produce the Ti4822-xNb alloy, where x = 1 at.%, 2 at.%, and 4 at.% designated as S1, S2, and S3, respectively.

**Figure 2:**
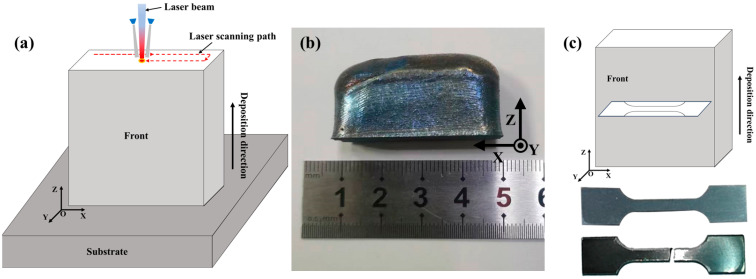
(**a**) Schematic of LMD processing, (**b**) photograph of as-built sample, and (**c**) sampling schematic and physical drawings of tensile samples.

Ti4822-xNb samples with a size of 35 mm × 10 mm × 35 mm were produced using an RC-LDM8060 machine (Yuchen Inc., Nanjing, China) with a 2000 W fiber laser and a six-channel powder feeding system. The LMD parameters used were a laser beam power of 1600 W, a beam-scanning speed of 7 mm·s^−1^, a layer thickness of 0.5 mm, and a beam diameter of 2.5 mm. An unalloyed Ti substrate was used for the deposition. High-purity argon gas was added to the printing chamber to keep the oxygen content below 50 ppm. A serpentine scanning strategy was adopted, as illustrated in Figure 2.

The specimens were ground, polished, and then etched with Kroll’s reagent (5 vol.% HF, 15 vol.% HNO_3_, and 80 vol.% by volume H_2_O) before examining the microstructure using a Quanta FEG250 SEM (FEI, Hillsboro, OR, USA) operated at 18 kV in the backscattered electron (BSE). The compositions of the points marked in BSE images were determined using X-ray energy dispersive spectroscopy (EDS, FEI, Hillsboro, OR, USA) at an accelerating voltage of 20 kV. The counting time for each point was 3 min. Electron backscatter diffraction (EBSD) examinations were performed at an operating voltage of 20 kV and a step size of 0.5 μm. Hardness tests were conducted using a Buehler5104 microhardness tester (Future Tech, Kanazawa, Japan) with a maximum indentation load of 0.3 kg and a duration of 20 s. Dog bone-shaped tensile specimens with gauges measuring 9.5 mm × 2 mm × 1.6 mm were cut parallel to the XY plane. Tensile tests were conducted at room temperature using a universal tensile machine (AGS-X, Shimadzu, Kyoto, Japan) with an initial strain rate of 1 × 10^−4^ s^−1^. Three samples were measured for each alloy, and the average was calculated. An HT-1000 friction and wear testing apparatus (Zhongke Kaihua Co., Ltd, Lanzhou, China) was used to conduct dry-sliding wear tests at room temperature. The samples were rotated by sliding against a 4 mm diameter Si_3_N_4_ ceramic ball (purchased from Sikeer Industry & Trade Co., Ltd, Kaifeng, China) for 30 min at a rotation speed of 560 r/min with a contact load of 1 kg. The wear marks on the samples after testing were observed and imaged using a VHX-5000 ultra depth of field 3D microscope system (Keyence Co,. Ltd., Shanghai, China).

## 3. Results and Discussion

This section will present and analyze the results of XRD, SEM, EBSD, tensile testing, and friction and wear testing for S1, S2, and S3 samples.

### 3.1. Microstructure

**Figure 3:**
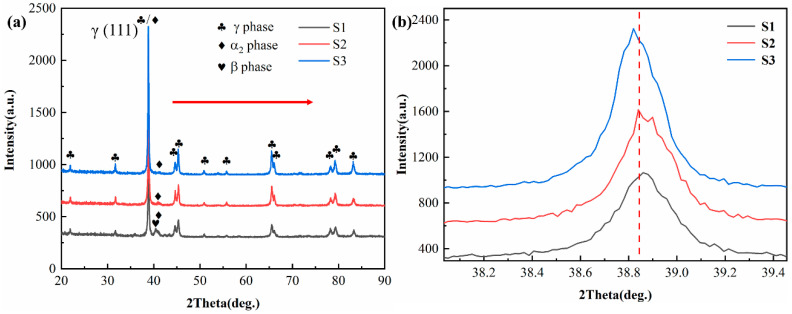
(**a**) XRD patterns of Ti4822-xNb alloys S1, S2 and S3, and (**b**) enlarged XRD patterns around the γ (111) peak.

XRD patterns revealed that the strongest diffraction peak from the Ti4822 powder corresponded to the α_2_ phase (201), see Figure 1b, indicating that the powder matrix was the α_2_ phase rather than the γ phase. This is because of the high cooling rate during powder processing. Figure 1c,d shows the morphology of the Ti4822 powder, which had good sphericity, dispersion, and surface finish, thereby meeting the requirements for LMD manufacturing. XRD patterns of S1, S2, and S3 are shown in Figure 3. The predominant diffraction peaks in all three samples corresponded to the γ phase (111). Most other diffraction peaks corresponded to the γ phase, while a few corresponded to the α_2_ phase, indicating that the samples were mostly composed of the γ phase with a small amount of α_2_ phase. Many X-ray peaks were bifurcated, because of the tetragonal crystal structure of the γ phases [38]. Note that S1 also had a diffraction peak corresponding to the β_0_ phase, which was not present in the other two samples. In addition, as the Nb content increased, the intensity of the diffraction peaks corresponding to the α_2_ phase decreased, while those corresponding to the γ phase increased, indicating reduced α_2_ content and increased γ content. Figure 3b shows an enlarged section of the XRD patterns around the γ (111) peak. It is evident that the diffraction peak corresponding to the (111) crystal plane of the γ phase shifted slightly to the left with increasing Nb, i.e., the 2*θ* value decreased. Thus, from Braggs law [48],

(1) 2dsin⁡θ=λ

where *d* is the crystal plane spacing, *θ* is the diffraction angle, and *λ* is the wavelength of the incident X-ray. A decrease in the value of *θ* implies an increase in the value of *d*, which indicates an increase in the lattice parameter of the γ phase. The lattice parameters increase when Nb replaces Ti in TiAl alloys because Nb has a larger atomic radius than both Ti and Al. Therefore, it is evident that the amount of Nb content in the γ phase increased.

**Figure 4:**
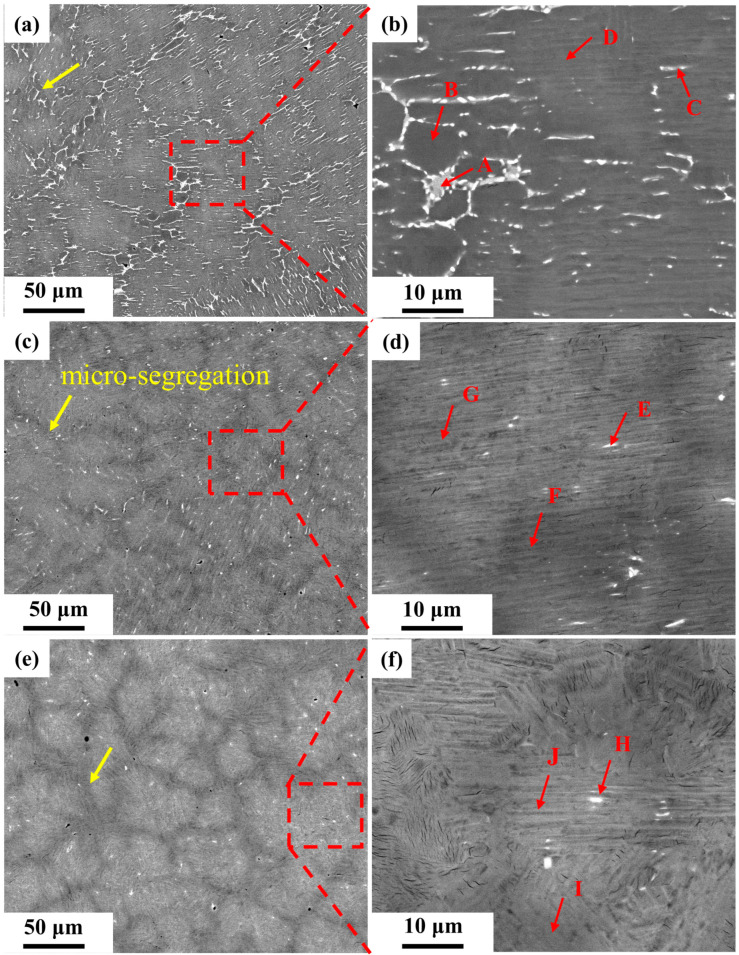
BSE images of Ti4822-xNb alloys (the red letters indicate features discussed in the text): (**a**) S1, (**b**) higher magnification image of the region in the red box in (**a**), (**c**) S2, (**d**) higher magnification image of the region in the red box in (**c**), (**e**) S3, (**f**) higher magnification image of the region in the red box in (**e**).

Figure 4 shows backscattered electron (BSE) images of the three LMDed Ti4822-xNb alloys. No unmelted Nb particles or significant Nb segregation were observed in the samples. This suggests that the Nb particles introduced as element powders were well melted and integrated during the deposition process. Due to its low atomic number, the Al will show dark contrast in BSE images. There were obvious black network bands in the samples (as yellow arrows designated), which were regions of segregation caused by the accumulation of Al, also known as S-segregation [49]. The β stabilazing elements have a higher atomic number, so the β_0_ phase appears bright white. The microstructures of all three samples exhibited S-segregation, with β_0_ phases dispersed throughout the matrix. As the Nb content increased, the S-segregation became more severe while the amount of the β_0_ phase decreased. To further clarify the phases present and their distribution, the chemical compositions of different regions of the samples were analyzed, see Table 1. The β_0_ phase in the three samples can be divided into two types. As shown in Figure 4b, the first type was larger and had a lower Al content (location A). The second type was finer and precipitated in the middle of the lamellae (locations C, E, and I in Figure 4b,d,f). Overall, Nb did not exhibit significant segregation, and the β_0_ phase was enriched in Cr. The degree of Cr enrichment in the β_0_ phase of S1 was notably greater than in S2 and S3.

**Figure 5:**
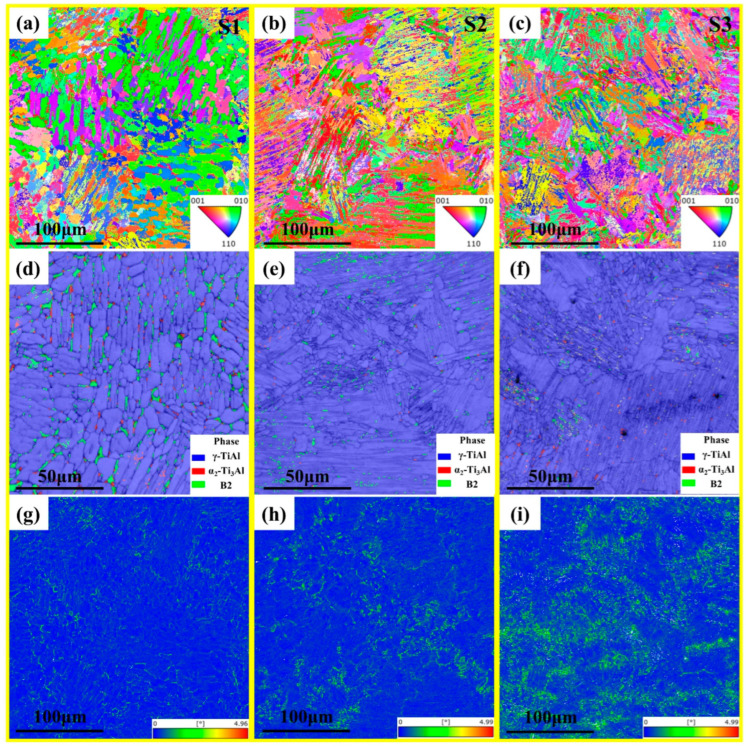
EBSD results including inverse pole figures (IPFs), phase maps, and kernel average misorientation (KAM) maps of Ti4822-xNb alloys: (**a**,**d**,**g**) S1, (**b**,**e**,**h**) S2, and (**c**,**f**,**i**) S3.

EBSD results from the three alloys are shown in Figure 5. From the inverse pole figures (Figure 5a–c), S1 exhibited a large number of massive grains, while S2 and S3 samples showed lamellar colonies. Note that the lamellar colony size of S3 was smaller than that of S2 because the greater Nb content refines the microstructure. There was no significant texture in any of the samples. Table 2 shows the volume fractions of the phase in the three alloys obtained from EBSD results. S1, S2, and S3 contained 92.8%, 97.7%, and 98.6% of the γ phase, respectively, the volume fractions of the β_0_ phase were 5.7%, 1.9%, and 1%, respectively, while the volume fractions of the α_2_ phase were 1.5%, 0.4%, and 0.4%, respectively. That the volume fraction of the β_0_ phase decreased with the increasing in Nb content is consistent with the BSE images. As shown in Figure 5d,e, the β_0_ phases in S1 were mainly located at the grain boundaries, whereas in S2 and S3, they were located in the middle of the lamellae. Furthermore, fine granular grains crystal clusters were present along the edges of the lamellar colonies, as illustrated in Figure 5e,f. Zhang et al. [38] and Wu et al. [28] also observed this phenomenon in Ti4822 alloys formed by LMD. This is because the laser repeatedly swept over the deposited material, causing the first deposited part to undergo solid phase transitions due to thermal cycling during LMD processing [28,37]. Figure 5g–i shows that the kernel average misorientation (KAM) of the samples increased with increasing Nb, which reflects the accumulation of local faults and stresses. A large amount of Nb in a solution causes lattice distortion in TiAl alloys, leading to stress concentrations. Additionally, Nb reduces the stacking fault energy of the γ phase and increases the tendency to form mechanical twins [50].

### 3.2. Microstructural Evolution Analysis

**Figure 6:**
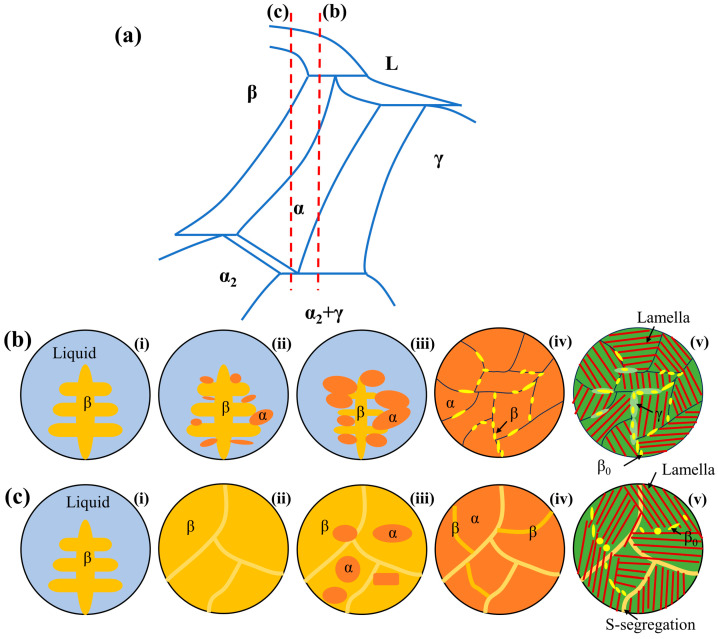
Schematic of the phase transformation in Ti4822-xNb alloys: (**a**) generalized Ti-Al binary phase diagram, (**b**) solidification pathway of S1, and (**c**) solidification pathways S2 and S3. (**b**,**c**) represent phase transformation during one cycle of LMD.

Based on the solidification pathways, TiAl alloys can be divided into two categories: peritectic-solidified TiAl alloys (PSG) and β-solidified TiAl alloys (BSG). Traditional PSG alloys typically have an Al content of over 45 at.% and experience a peritectic reaction (L + β→α) during the cooling process [51]. Ti4822 is a typical PSG alloy. The above results indicate that the microstructure of S1 significantly differed from those of S2 and S3, probably due to its different solidification path. The chemical composition of S1 was closely similar to that of the base Ti4822 alloy, leading to the peritectic solidification. Figure 6a depicts the corresponding microstructural evolution of peritectic solidification of the TiAl alloy. During the LMD process, the powders were irradiated by a high-energy laser beam, leading to a local temperature spike instantaneously above the melting point. A micro-scale molten pool was created in a short period. The primary β phase nucleated and grew from the molten liquid phase. Subsequently, the α phase formed on the surface of the primary β phase because of the lower energy of nucleation and consumed both the liquid and β phases during growth. Simultaneously, the diffusion of Al from the β phase into the α phase occurred, while β-stabilizing elements diffused into the β phase. This trend led to an enrichment of β-stabilizing elements and an absence of Al at the junction of β and α grains. The cooling rate was so rapid that the alloy underwent non-equilibrium solidification, resulting in β_0_ (location A in Figure 4b) + γ (location B in Figure 4b) formation. The primary β phase is a heterogeneous nucleation site which enhances the nucleation rate and generates numerous refined α grains [52]. The β_0_ phase at the grain boundary is conducive to limiting the growth of α grains during subsequent thermal cycles. If α grains have an opportunity to coarsen afterward, the segregation of the β-stabilizing elements is transferred to the inner lamellae of the grains, see location C in Figure 4b.

Both the alloying elements and the cooling rate during solidification will affect the solidification path [11]. The larger Nb addition in the S2 and S3 samples further expanded the β field area to encompass higher levels of Al content, resulting in the occurrence of β-solidification, as illustrated in Figure 6b. The presence of significant S-segregation (Figure 4c,e) in both microstructures provides compelling evidence for this. The phase transition passes through a single-phase β field, which leads to the solidification of the β phase first and pushes the Al to the grain boundaries, producing S-segregation. At the phase transformation point of β→α + β, the β-stabilizing elements are expelled from both the boundary and the α/β interface. When the transition from β to α is incomplete, the segregation site tends to form a β_0_ phase, which is dependent on the Al content present [49]. As previously described, the laser rapidly melts the powders to form a miniature molten pool. However, due to the short time, the Nb powders tend to form atomic clusters after melting [53]. On the one hand, Nb can increase the liquidus temperature of TiAl alloys, thereby promoting nucleation in the Nb-rich region. On the other hand, the Nb atomic clusters can act as heterogeneous sites to promote nucleation, thus refining the primary β grains. Consequently, in comparison to S2, S3 had finer lamellar colonies, although this also leads to a more severe S-segregation.

The above results show that the Nb element is less prone to segregation during LMD processing, which can be ascribed to the high cooling and the low diffusion coefficient. The segregation of Cr is the reason for the residual β_0_ phase at room temperature. Adding Nb can alter the solidification path of the LMD-ed TiAl alloys, which can consequently affect their microstructures. Compared to the peritectic solidification path (S1), β-solidification (S2 and S3) is advantageous for reducing Cr segregation. This is because compared to the solid phase, the solute diffuses faster in the liquid phase [54]. Additionally, a significant amount of Nb in a solid solution can decrease the diffusion efficiency rate of Cr atoms.

### 3.3. Mechanical Properties

**Figure 7:**
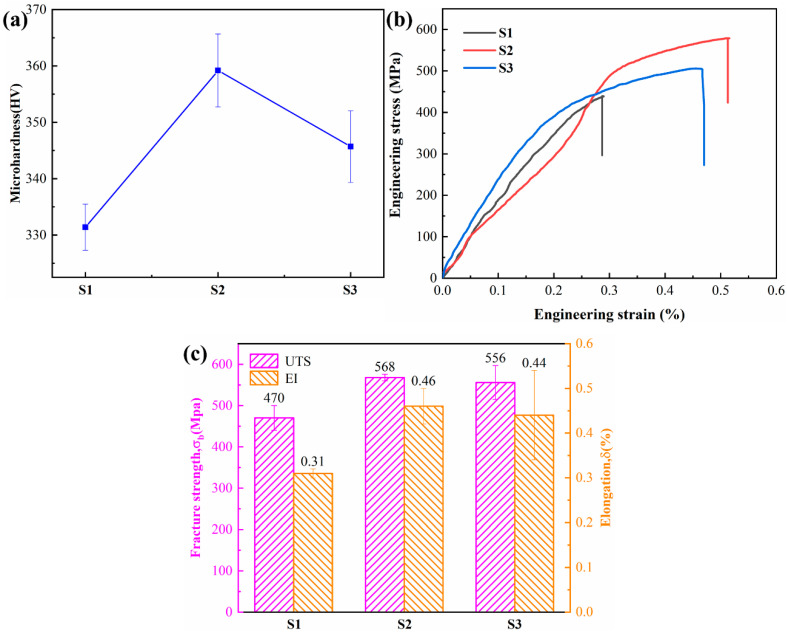
Mechanical properties of Ti4822-xNb alloys: (**a**) microhardness, (**b**) RT tensile engineering stress-strain curve, and (**c**) RT tensile strength and elongation.

The microhardness results for S1, S2, and S3 are shown in Figure 7a. Five points were measured for each. The microhardness first increased and then decreased with increasing Nb content, with the S2 sample having the highest microhardness of 359 ± 6 HV0.3. Typical RT tensile engineering stress–strain curves are presented in Figure 7b, and the tensile results (which are the average of three tests) are presented in Figure 7c. Compared to S1, whose fracture strength was 470 ± 30 MPa, S2 exhibited an increase of nearly 100 MPa, reaching 568 ± 8 MPa. The fracture strengths of S2 and S3 samples were similar, indicating that the additional Nb did not effectively improve the strength. All three samples showed brittle fracture with elongations less than 0.5%.

**Figure 8:**
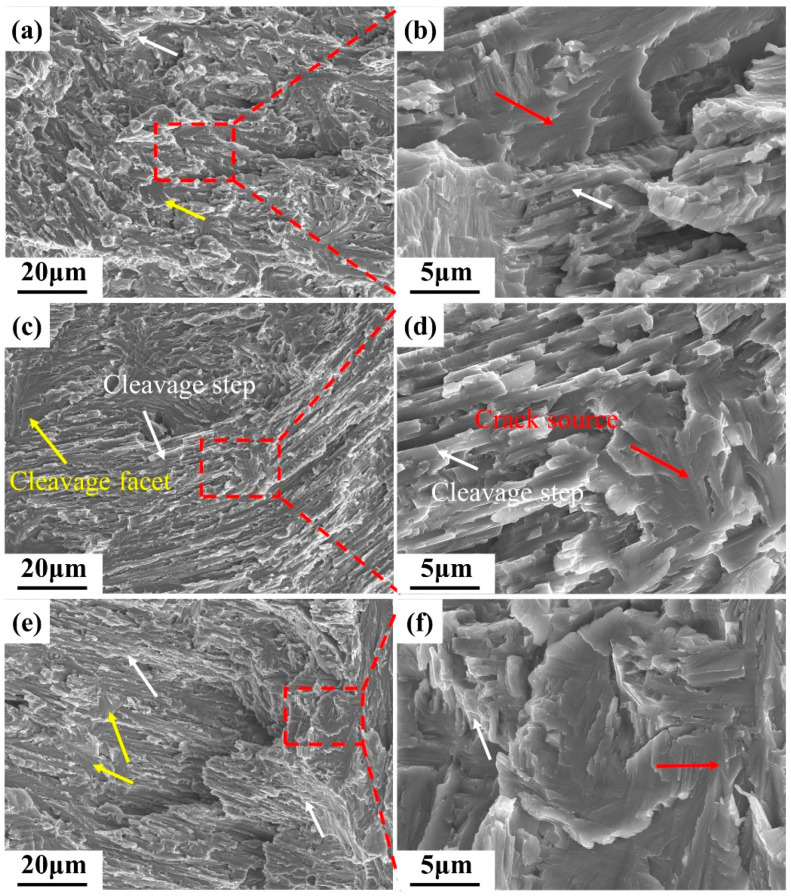
SEM results of fracture surfaces of Ti4822-xNb alloys (yellow arrows indicate cleavage facets; white arrows indicated cleavage steps; red arrows indicated the source of the cracks): (**a**) S1, (**b**) higher magnification image of the region in the red box in (**a**), (**c**) S2, (**d**) higher magnification image of the region in the red box in (**c**), (**e**) S3, and (**f**) higher magnification image of the region in the red box in (**e**).

Figure 8 shows the fracture surfaces of the three alloys after tensile testing. All the alloys exhibited brittle fracture behaviors, as evidenced by the cleavage facets. In Figure 8b,d,f, point-radiant trans-granular expansion (the red arrows) across multiple lamella thicknesses can be found. According to the fracture theory, the point of expansion is the source of cracks.

The mechanical properties of the TiAl alloys depend on two factors. First, Nb is present in a solid solution in the TiAl matrix. This not only alters the phase transformation interval but also strengthens the alloy. The addition of Nb increases the c/a ratio of the γ phase, makes it more anisotropic, and increases the strength of the γ phase [44]. Therefore, the hardness and strength of S2 and S3 were greater than those of S1. However, increasing the Nb content resulted in slightly lower hardness values for S3. This is because lattice distortion increases the brittleness of the alloys. Second, the mechanical properties are significantly affected by the phases present [55]. The γ phase has a face-centered tetragonal structure with many independent slip systems. The α_2_ phase has a close-packed hexagonal structure, and its slip system is limited. The β_0_ phase, which has an ordered body-centered cubic structure, is a hard and brittle phase at room temperature and is detrimental to the ductility. Therefore, the improved properties of the S2 sample also benefited from the increment of the γ phase and the reduction in the β_0_ phase.

### 3.4. Tribological Properties

**Figure 9:**
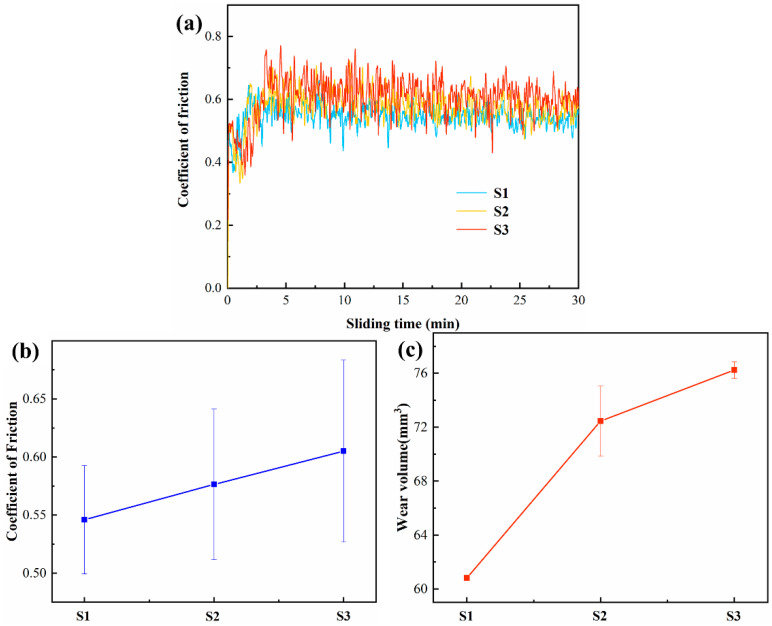
(**a**) Friction coefficient-sliding time curves, (**b**) average friction coefficients, and (**c**) wear volumes for the Ti4822-xNb alloys.

**Figure 10:**
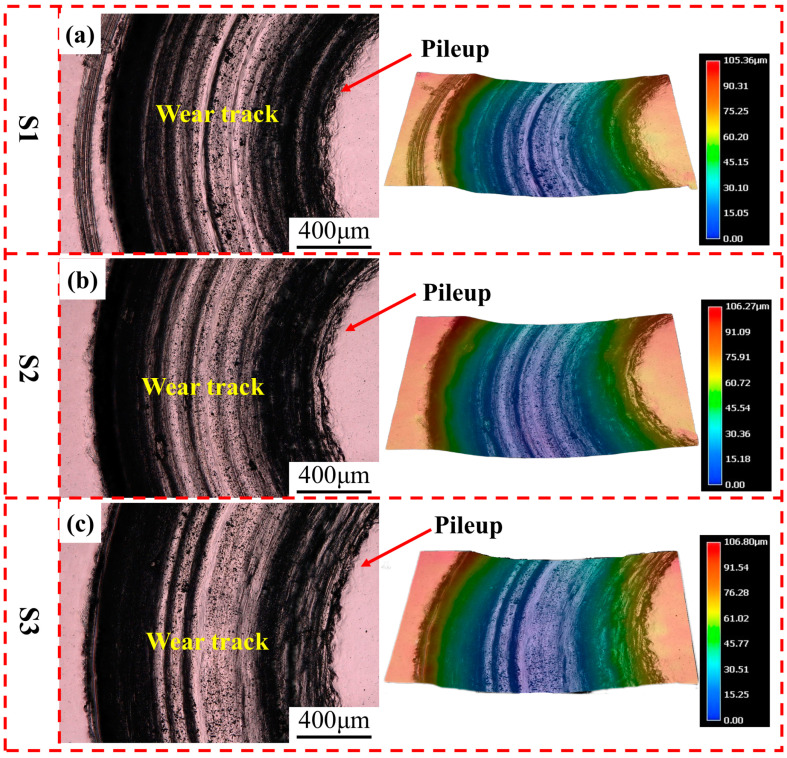
Surface observation and 3D images from ultra-depth three-dimensional microscope for wear tracks of Ti4822-xNb alloys: (**a**) S1, (**b**) S2, and (**c**) S3.

Figure 9a displays curves for the coefficient of friction for the alloys during wear testing in air under a 10 N normal load. The coefficient of friction initially increased with increasing sliding time and stabilized after ~5 min. In the initial run-in period, the actual contact area was small, resulting in a low friction coefficient [56]. After a period of running, the contact area gradually increased as materials wore away. During the stable stage, the friction coefficients of the three samples still fluctuated somewhat, but the fluctuations decreased with time. Figure 9b shows the average friction coefficients for S1, S2, and S3 as 0.55 ± 0.05, 0.58 ± 0.06, and 0.61 ± 0.08, respectively. The errors of the friction coefficient reflect the degree of fluctuation. Both the friction coefficient and the fluctuations slightly increased as the Nb content rose. The wear volume, V, was calculated from the following:

(2) V=2πR·S

where *R* is the radius of the test and *S* is the cross-sectional area of the wear track. The radius of the abrasion paths was measured, and the values of *S* were determined using the ultra-depth-of-field three-dimensional microscopic system, as shown in Figure 9. The resulting wear volumes for S1, S2, and S3 were 60.8 ± 0.1 mm^3^, 72.5 ± 2.6 mm^3^, and 76.2 ± 0.6 mm^3^, respectively, see Figure 9c, i.e., the wear volume increased as the Nb content increased. Figure 10 clearly shows that there was a pileup of material at the boundary between the track and the unworn area, indicating the existence of local plastic deformation in all the alloys. It has been demonstrated that, in the absence of any surface irregularities, the maximum shear stress under the surface of a rigid sphere pressed against a flat material can be up to 0.48 times the contact stress [57]. However, in practice, surface roughness necessitates considering additional factors, resulting in a three times higher accurate shear stress level [58]. When a normal load of 10 N is applied, the normal surface contact stress is approximately 1000 MPa, corresponding to a sub-surface shear stress of over 1400 MPa. This is considerably higher than the yield strength of Ti4822-xNb alloys [59]. As a result, plastic deformation occurs during wear testing, leading to a pile-up at the edge of the wear track.

**Figure 11:**
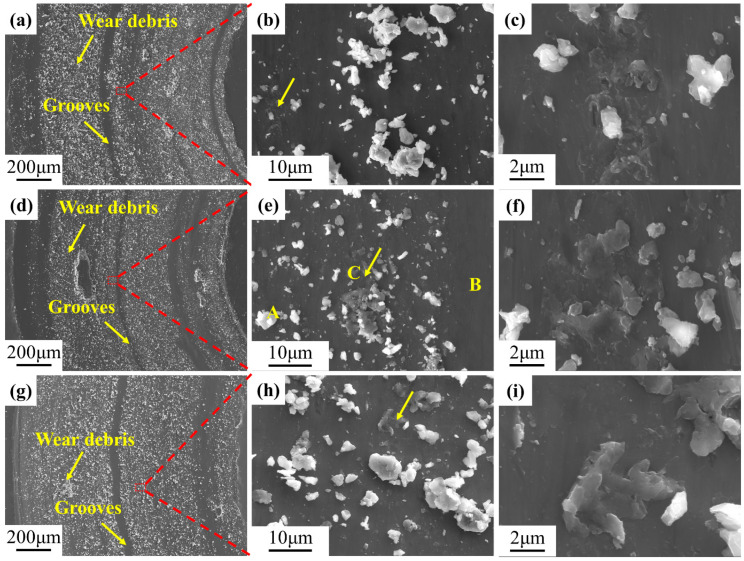
Secondary electron images showing the wear surface of Ti4822-xNb alloys and corresponding EDS maps: (**a**) S1, (**b**) a higher magnification image of the region in the red box in (**a**), (**c**) a higher magnification image of the region indicated by the yellow arrow in (**b**), (**d**) S2, (**e**) a higher magnification image of the region in the red box in (**d**) (the yellow letters indicate different characteristic areas), (**f**) a higher magnification image of the region indicated by the yellow arrow in (**e**), (**g**) S3, (**h**) a higher magnification image of the region in the red box in (**g**), (**i**) a higher magnification image of the region indicated by the yellow arrow in (**h**).

To analyze the wear mechanisms of the alloys, the wear tracks of samples were examined further. Figure 11 shows secondary electron images. The wear surfaces of the three samples were similar, and the wear tracks were generally relatively flat. The surface of the wear tracks contained a significant amount of white and bright particles, which were wear debris [60]. The surface damage consisted of tens of micrometers wide and long continuous plowed grooves. This indicates that the primary wear mechanism of TiAl alloy is abrasive wear, which is consistent with reports in the literature [7,61,62]. Upon further magnification, gray “scars” (the yellow arrows in Figure 11b,e,h) could been seen were present on the wear surfaces, which were clearly indications of plastic deformation. Figure 11c,f,i shows the scars in detail. In the S2 sample, three typical morphologies were analyzed: wear debris, matrix, and scar. Point analyses were carried out at A, B, and C in Figure 11, and the results are presented in Table 3. The results revealed that wear debris showed a high O content of 31.7%, which indicates that oxidative wear occurred. The matrix contained only a small amount of O, suggesting that no tribo-oxide layer formed on the wear surfaces. The O content at the scar was between matrix and wear debris. All three sites contained only trace amounts of Si.

**Figure 12:**
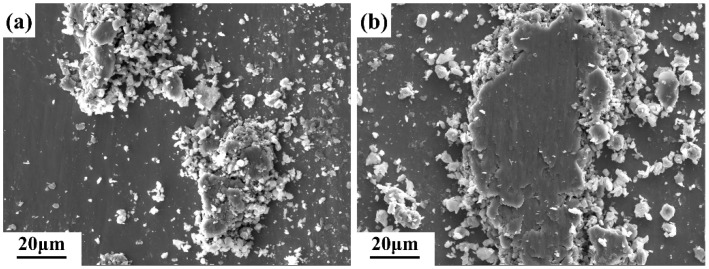
Secondary electron images showing (**a**) agglomeration and (**b**) compaction of wear debris.

As previously stated, the surface of the samples was subjected to considerably elevated stresses compared to its yield strength during friction, which resulted in plastic deformation of the asperities (Figure 11c,f,i). Upon reaching a certain threshold, plastic deformation ceded to the generation of wear debris. Some of the wear debris was expelled from the wear testing system under the force of centrifugal acceleration. At the same time, the rest remained between the wearing components, continuing to contribute to the wear process. The heat generation by friction resulted in an elevated local temperature, which stimulated the fine abrasive particles to react with oxygen in the air to form metal oxide particles (Table 3). The action of both frictional and normal stresses led to the agglomeration, compaction, and welding of these metal oxides, as shown in Figure 12. Nevertheless, a friction oxide layer with good wear resistance was not formed further. Consequently, the wear process became more intense, and the coefficient of friction exhibited considerable fluctuations.

**Figure 13:**
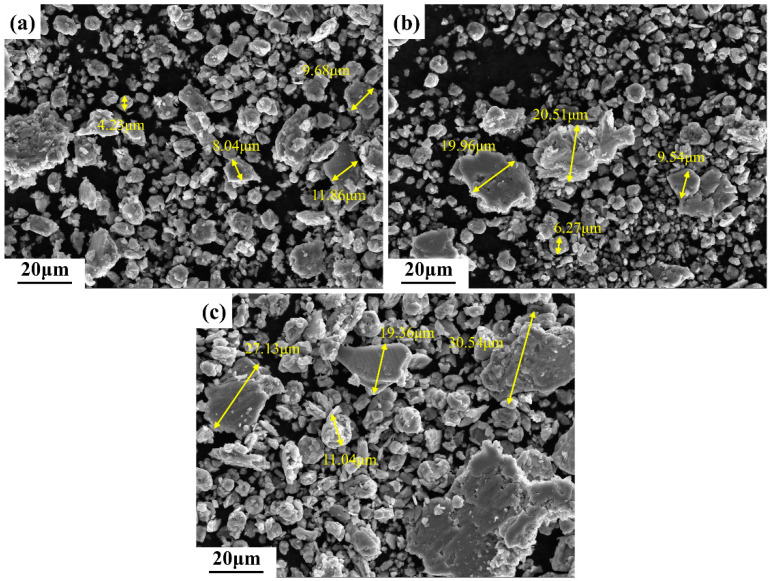
Secondary electron images showing the wear debris from (**a**) S1, (**b**) S2, and (**c**) S3.

In the presence of a load, the Si_3_N_4_ ceramic ball was capable of exerting pressure on the surface of the alloy samples, resulting in the material undergoing flow. In this context, the role of shear stress was of particular significance. It has been demonstrated that the γ/γ and α_2_/γ interfaces in TiAl alloys exert a significant blocking effect on the sliding of the anti-friction material [46]. In comparison to S1, S2 and S3 contained a considerable number of lamellar interfaces. Further, the increased Nb content resulted in a more severe lattice distortion of the γ phase, necessitating a higher shear stress for material flow. This manifested itself macroscopically as an increase in the coefficient of friction. However, the wear volume of the three samples showed an opposite trend. The microstructure is the primary factor influencing the tribological behavior under identical sliding conditions. S1 exhibited a microstructure with more β_0_ phases (5.7%) than the other two alloys (1–1.9%) and thus demonstrated better plasticity and toughness under local high-temperature conditions, which may reduce the wear and plowing action of Si_3_N_4_ and oxide particles. Furthermore, the increase in Nb content resulted in a more pronounced S-segregation in the alloys, accompanied by a reduction in microstructural homogeneity. As previously stated, the lamellar colonies of S3 were smaller and more numerous than those of S2. Additionally, delicate γ grain clusters were present at the boundaries of the lamellar colonies of both. It has been reported in the literature that the hardness of lamellar structures in TiAl alloys is higher than that of massive or equiaxed γ grains [28]. The hardness of a material reflects the ability to resist deformation or damage to its surface. When subjected to shear stress, the uneven distribution of strain between structures with different hardness values leads to the initiation of cracks. The morphologies of the abrasive debris in all three samples can be observed in Figure 13. As the Nb content increased, the size of abrasive debris also increased, particularly in S3. The heterogeneous composition and structure of the alloy rendered it susceptible to the detachment of large pieces during friction, which in turn resulted in an augmented rate of wear. This is consistent with the preceding analysis. Therefore, the wear performance of the alloy diminished with the increase in Nb content.

## 4. Conclusions

In this work, Ti4822 alloys with different additional Nb contents were successfully prepared using LMD processing. The microstructure, mechanical, and tribological properties of the as-fabricated TiAl alloys were characterized and analyzed. The following conclusions can be drawn:

1. The TiAl alloys prepared by LMD showed a decrease in the β_0_ phase and an increase in the γ phase with increasing Nb content. The presence of the residual β_0_ phase was primarily caused by Cr segregation and weakly correlated with the Nb content. Conversely, the increase in Nb altered the solidification path of TiAl alloys from peritectic solidification to β-solidification. The increase in Nb content obviously refined the microstructures and increased the tendency for S-segregation.
2. As the Nb content increased, the microhardness of the sample initially increased and then decreased with the alloy containing an additional 2 at.% Nb exhibiting the highest hardness value of 359.2 ± 6.5 HV_0.3_. Furthermore, this alloy exhibited a fracture strength of 568 ± 7.8 MPa, which was nearly 100 MPa higher than that of the modified alloy with an additional 1 at. % Nb. The alloy with more Nb (4 at.%) showed the same strength as that with an additional 2 at.%.
3. The wear resistance of the as-fabricated alloys decreased with increasing Nb content. The main wear mechanism for all alloys was abrasive wear, while oxidative wear occurred without the formation of tribo-oxide layers. In addition, local plastic deformation and pile-ups at the edges of the wear tracks occurred.

## Figures and Tables

**Table 1:** EDS results of the marked areas in Figure 4.

Label	Chemical Composition (at.%)				Identified Phase
	Ti	Al	Nb	Cr	
A	54.1	35.6	2.0	8.3	β_0_
B	44.1	52.5	2.2	1.2	γ
C	48.4	46.2	2.5	3.0	β_0_
D	47.7	47.8	2.6	1.9	γ/α_2_
E	45.9	46.5	4.6	3.0	β_0_
F	44.7	49.6	3.3	2.4	γ/α_2_
G	45.8	47.8	4.3	2.0	γ/α_2_
H	44.8	47.4	4.1	3.7	β_0_
I	44.8	49.4	3.7	2.1	γ/α_2_
J	45.4	48.0	4.7	1.9	γ/α_2_

**Table 2:** Volume fractions of phases in S1, S2, and S3 from EBSD results.

Alloys	Phase Content (%)		
	γ-TiAl	β_0_/B2	α_2_-Ti_3_Al
S1	92.8	5.7	1.5
S2	97.7	1.9	0.4
S3	98.6	1	0.4

**Table 3:** EDS results from the marked areas in Figure 11e.

Label	Composition in at.%					
	Ti	Al	Nb	Cr	O	Si
A	31.4	32.7	2.8	1.2	31.7	0.1
B	46.9	43.1	3.5	2.0	4.3	0.2
C	40.7	39.1	3.2	1.7	15.2	0.1

## Data Availability

The data presented in this study are available on request from the corresponding author.

## References

[1] Clemens H., Mayer S. (2013). Design, Processing, Microstructure, Properties, and Applications of Advanced Intermetallic TiAl Alloys. Adv. Eng. Mater..

[2] Lu Q., Xu Z., Chen X., Feng P., Li C., Yi J. (2024). Evading the strength and ductility trade-off dilemma in titanium matrix composites through supersaturated solid solution and phase transformation. Mater. Sci. Eng. A.

[3] Park J.-S., Yang G., Kim S.-W. (2023). A high tensile strength above 900 °C in β-solidified TiAl alloy through alloy design and microstructure optimization. J. Alloys Compd..

[4] Zheng G., Tang B., Zhao S., Wang J., Xie Y., Chen X., Wang W.Y., Liu D., Yang R., Li J. (2023). Breaking the high-temperature strength-ductility trade-off in TiAl alloys through microstructural optimization. Int. J. Plast..

[5] Gao P., Huang W., Yang H., Jing G., Liu Q., Wang G., Wang Z., Zeng X. (2020). Cracking behavior and control of β-solidifying Ti-40Al-9V-0.5Y alloy produced by selective laser melting. J. Mater. Sci. Technol..

[6] Cho K., Kobayashi R., Oh J.Y., Yasuda H.Y., Todai M., Nakano T., Ikeda A., Ueda M., Takeyama M. (2018). Influence of unique layered microstructure on fatigue properties of Ti-48Al-2Cr-2Nb alloys fabricated by electron beam melting. Intermetallics.

[7] Lu X., Li J., Chen X., Qiu J., Wang Y., Liu B., Liu Y., Rashad M., Pan F. (2020). Mechanical, tribological and electrochemical corrosion properties of in-situ synthesized Al_2_O_3_/TiAl composites. Intermetallics.

[8] Liu S., Ding H., Chen R., Guo J., Fu H. (2021). Evolution of rapidly grown cellular microstructure during heat treatment of TiAl-based intermetallic and its effect on micromechanical properties. Intermetallics.

[9] Wang J., Pan Z., Wei L., He S., Cuiuri D., Li H. (2019). Introduction of ternary alloying element in wire arc additive manufacturing of titanium aluminide intermetallic. Addit. Manuf..

[10] Liang Y.-F., Xu X.-J., Lin J.-P. (2016). Advances in phase relationship for high Nb-containing TiAl alloys. Rare Met..

[11] Duan B., Yang Y., He S., Feng Q., Mao L., Zhang X., Jiao L., Lu X., Chen G., Li C. (2022). History and development of γ-TiAl alloys and the effect of alloying elements on their phase transformations. J. Alloys Compd..

[12] Yu J., Lin X., Wang J., Chen J., Huang W. (2010). Mechanics and energy analysis on molten pool spreading during laser solid forming. Appl. Surf. Sci..

[13] Schwaighofer E., Clemens H., Mayer S., Lindemann J., Klose J., Smarsly W., Güther V. (2014). Microstructural design and mechanical properties of a cast and heat-treated intermetallic multi-phase γ-TiAl based alloy. Intermetallics.

[14] Luo Y., Liu S., Sun Z., Liu B., Wang L., Wang Y., Liu Y. (2023). Microstructural evolution during annealing of a powder metallurgical TiAl-Nb composite and its effect on mechanical properties. J. Mater. Res. Technol..

[15] Schloffer M., Iqbal F., Gabrisch H., Schwaighofer E., Schimansky F.-P., Mayer S., Stark A., Lippmann T., Göken M., Pyczak F. (2012). Microstructure development and hardness of a powder metallurgical multi phase γ-TiAl based alloy. Intermetallics.

[16] Zhang S., Tian N., Li J., Yang G., Yang W., Wang G., Liu Z., Li Y. (2023). Microstructure evolution of a forged TiAl-Nb alloy during high-temperature tensile testing. Mater. Charact..

[17] Zhang S.Z., Zhao Y.B., Zhang C.J., Han J.C., Sun M.J., Xu M. (2017). The microstructure, mechanical properties, and oxidation behavior of beta-gamma TiAl alloy with excellent hot workability. Mater. Sci. Eng. A.

[18] Liu S., Ding H., Zhang H., Chen R., Guo J., Fu H. (2018). High-density deformation nanotwin induced significant improvement in the plasticity of polycrystalline γ-TiAl-based intermetallic alloys. Nanoscale.

[19] Li H., Qi Y., Liang X., Zhu Z., Lv F., Liu Y., Yang Y. (2016). Microstructure and high temperature mechanical properties of powder metallurgical Ti-45Al-7Nb-0.3W alloy sheets. Mater. Des..

[20] Ren Y., Han B., Wu H., Wang J., Liu B., Wei B., Jiao Z., Baker I. (2023). Copper segregation-mediated formation of nanotwins and 9R phase in titanium alloys produced by laser powder bed fusion. Scr. Mater..

[21] Zhang T., Liu C.-T. (2022). Design of titanium alloys by additive manufacturing: A critical review. Adv. Powder Mater..

[22] Xue H., Liu C., Song Y., Liang Y., Tong X., Wang Y., Lin J. (2023). Additive manufacturing of nano-W composite high Nb-TiAl alloys fabricated via selective laser melting. Mater. Lett..

[23] Liu M., Wang J., Hu T., Xu S., Shuai S., Xuan W., Yin S., Chen C., Ren Z. (2024). Laser powder bed fusion of a Ni3Al-based intermetallic alloy with tailored microstructure and superior mechanical performance. Adv. Powder Mater..

[24] Sun J.e., Zhang B., Qu X. (2021). High strength Al alloy development for laser powder bed fusion. J. Micromech. Mol. Phys..

[25] DebRoy T., Wei H.L., Zuback J.S., Mukherjee T., Elmer J.W., Milewski J.O., Beese A.M., Wilson-Heid A., De A., Zhang W. (2018). Additive manufacturing of metallic components—Process, structure and properties. Prog. Mater Sci..

[26] Xue H., Liang Y., Peng H., Wang Y., Shang S.-L., Liu Z.-K., Lin J. (2023). An additively manufactured γ-based high Nb-TiAl composite via coherent interface regulation. Scr. Mater..

[27] Zhu Y., Wang Z., Yu B., Li G., Xue Y., Liang Y.-J. (2023). Additive manufacturing of fine-grain fully lamellar titanium aluminide alloys. Mater. Des..

[28] Wu Y., Zhang S., Cheng X., Wang H. (2019). Investigation on solid-state phase transformation in a Ti-47Al-2Cr-2V alloy due to thermal cycling during laser additive manufacturing process. J. Alloys Compd..

[29] Wang D., Yang Y., Wang Y., Yang L., Wang H., Yang S. (2021). Introduction to the Special Issue on Design and Simulation in Additive Manufacturing. Cmes-Comput. Model. Eng. Sci..

[30] Cao L. (2021). Mesoscopic-Scale Numerical Investigation Including the Influence of Process Parameters on LPBF Multi-Layer Multi-Path Formation. Cmes-Comput. Model. Eng. Sci..

[31] Liu Z., Zhao D., Wang P., Yan M., Yang C., Chen Z., Lu J., Lu Z. (2022). Additive manufacturing of metals: Microstructure evolution and multistage control. J. Mater. Sci. Technol..

[32] Zhang X., Li C., Wu M., Ye Z., Wang Q., Gu J. (2022). Atypical pathways for lamellar and twinning transformations in rapidly solidified TiAl alloy. Acta Mater..

[33] Sui S., Chew Y., Hao Z., Weng F., Tan C., Du Z., Bi G. (2022). Effect of cyclic heat treatment on microstructure and mechanical properties of laser aided additive manufacturing Ti–6Al–2Sn–4Zr–2Mo alloy. Adv. Powder Mater..

[34] Fan H., Liu Y., Yang S. (2021). Martensite decomposition during post-heat treatments and the aging response of near-α Ti–6Al–2Sn–4Zr–2Mo (Ti-6242) titanium alloy processed by selective laser melting (SLM). J. Micromech. Mol. Phys..

[35] Sharman A., Hughes J., Ridgway K. (2018). Characterisation of titanium aluminide components manufactured by laser metal deposition. Intermetallics.

[36] Zheng G., Tang B., Chen W., Zhao S., Xie Y., Chen X., Li J., Zhu L. (2023). Long-period stacking ordering induced ductility of nanolamellar TiAl alloy at elevated temperature. Mater. Res. Lett..

[37] Wang J., Luo Q., Wang H., Wu Y., Cheng X., Tang H. (2020). Microstructure characteristics and failure mechanisms of Ti-48Al-2Nb-2Cr titanium aluminide intermetallic alloy fabricated by directed energy deposition technique. Addit. Manuf..

[38] Zhang X., Li C., Zheng M., Ye Z., Yang X., Gu J. (2020). Anisotropic tensile behavior of Ti-47Al-2Cr-2Nb alloy fabricated by direct laser deposition. Addit. Manuf..

[39] Fatoba O.S., Lasisi A.M., Ikumapayi O.M., Akinlabi S.A., Akinlabi E.T. (2021). Computational modelling of laser additive manufactured (LAM) Titanium alloy grade 5. Mater. Today Proc..

[40] Shi X., Wang H., Feng W., Zhang Y., Ma S., Wei J. (2020). The crack and pore formation mechanism of Ti–47Al–2Cr–2Nb alloy fabricated by selective laser melting. Int. J. Refract. Met. Hard Mater..

[41] Chlupová A., Heczko M., Obrtlík K., Polák J., Roupcová P., Beran P., Kruml T. (2016). Mechanical properties of high niobium TiAl alloys doped with Mo and C. Mater. Des..

[42] Xu R., Li M., Zhao Y. (2023). A review of microstructure control and mechanical performance optimization of γ-TiAl alloys. J. Alloys Compd..

[43] Tetsui T. (2002). Effects of high niobium addition on the mechanical properties and high-temperature deformability of gamma TiAl alloy. Intermetallics.

[44] Liu Z.C., Lin J.P., Li S.J., Chen G.L. (2002). Effects of Nb and Al on the microstructures and mechanical properties of high Nb containing TiAl base alloys. Intermetallics.

[45] Fang H., Chen R., Liu Y., Tan Y., Su Y., Ding H., Guo J. (2019). Effects of niobium on phase composition and improving mechanical properties in TiAl alloy reinforced by Ti_2_AlC. Intermetallics.

[46] Wang H., Wang Q., Zeng L., Zhang H., Ding H. (2021). Microstructure, mechanical and tribological performances of a directionally solidified γ-TiAl alloy. Mater. Charact..

[47] Cheng J., Yang J., Zhang X., Zhong H., Ma J., Li F., Fu L., Bi Q., Li J., Liu W. (2012). High temperature tribological behavior of a Ti-46Al-2Cr-2Nb intermetallics. Intermetallics.

[48] Wang Q., Ding H., Zhang H., Chen R., Guo J., Fu H. (2018). Influence of Mn addition on the microstructure and mechanical properties of a directionally solidified γ-TiAl alloy. Mater. Charact..

[49] Chen G.L., Xu X.J., Teng Z.K., Wang Y.L., Lin J.P. (2007). Microsegregation in high Nb containing TiAl alloy ingots beyond laboratory scale. Intermetallics.

[50] Zhang W.J., Liu Z.C., Chen G.L., Kim Y.W. (1999). Deformation mechanisms in a high-Nb containing γ–TiAl alloy at 900 °C. Mater. Sci. Eng. A.

[51] Nath P., Bar H.N., Bhattacharjee A., Sen I. (2024). Designing of novel microstructure and its impact on the improved service temperature mechanical performance of 2nd and 3rd generation advanced intermetallic TiAl alloys. Mater. Sci. Eng. A.

[52] Wang Q., Liu X., Ren Y., Song M., Baker I., Wu H. (2024). Microstructural evolution and cryogenic and ambient temperature deformation behavior of the near-α titanium alloy TA15 fabricated by laser powder bed fusion. J. Alloys Compd..

[53] He P., Webster R.F., Yakubov V., Kong H., Yang Q., Huang S., Ferry M., Kruzic J.J., Li X. (2021). Fatigue and dynamic aging behavior of a high strength Al-5024 alloy fabricated by laser powder bed fusion additive manufacturing. Acta Mater..

[54] Singh V., Mondal C., Sarkar R., Bhattacharjee P.P., Ghosal P. (2021). Effects of Cr alloying on the evolution of solidification microstructure and phase transformations of high-Nb containing γ-TiAl based alloys. Intermetallics.

[55] Fang H., Chen R., Chen X., Yang Y., Su Y., Ding H., Guo J. (2019). Effect of Ta element on microstructure formation and mechanical properties of high-Nb TiAl alloys. Intermetallics.

[56] Agbedor S.-O., Wu H., Ren Y., Liang L., Yang D., Liu B., Liu Y., Baker I. (2024). A two-decade odyssey in fusion-based additive manufacturing of titanium alloys and composites. Appl. Mater. Today.

[57] Ovcharenko A., Halperin G., Verberne G., Etsion I. (2007). In situ investigation of the contact area in elastic-plastic spherical contact during loading-unloading. Tribol. Lett..

[58] Rastkar A.R., Bloyce A., Bell T. (2000). Sliding wear behaviour of two gamma-based titanium aluminides. Wear.

[59] Wang Q., Ding H., Zhang H., Chen R., Guo J., Fu H. (2017). Variations of microstructure and tensile property of γ-TiAl alloys with 0–0.5 at% C additives. Mater. Sci. Eng. A-Struct. Mater. Prop. Microstruct. Process..

[60] Du J., Ren Y., Liu X., Xu F., Wang X., Zhou R., Baker I., Wu H. (2023). Microstructural Evolution, Mechanical Properties and Tribological Behavior of B(4)C-Reinforced Ti In Situ Composites Produced by Laser Powder Bed Fusion. Materials.

[61] Cheng F., Lin J., Liang Y. (2019). Friction and wear properties of a high Nb-containing TiAl alloy against WC-8Co, Si_3_N_4_, and GCr15 in an unlubricated contact. Intermetallics.

[62] Zhou H., Su Y., Liu N., Kong F., Wang X., Zhang X., Chen Y. (2018). Modification of microstructure and properties of Ti-47Al-2Cr-4Nb-0.3W alloys fabricated by SPS with trace multilayer graphene addition. Mater. Charact..

